# Neutralising capacity against Delta (B.1.617.2) and other variants of concern following Comirnaty (BNT162b2, BioNTech/Pfizer) vaccination in health care workers, Israel

**DOI:** 10.2807/1560-7917.ES.2021.26.26.2100557

**Published:** 2021-07-01

**Authors:** Yaniv Lustig, Neta Zuckerman, Ital Nemet, Nofar Atari, Limor Kliker, Gili Regev-Yochay, Einav Sapir, Orna Mor, Sharon Alroy-Preis, Ella Mendelson, Michal Mandelboim

**Affiliations:** 1 Central Virology Laboratory, Public Health Services, Ministry of Health and Sheba Medical Center, Tel Hashomer, Israel; 2 Sackler Faculty of Medicine, Tel Aviv University, Tel Aviv, Israel; 3 Sheba Medical Center, Tel Hashomer, Israel; 4 Public Health Services, Ministry of Health, Jerusalem, Israel

**Keywords:** COVID-19, BNT162b2 vaccination, neutralizing, variants, B.1.617.2 (Delta)

## Abstract

SARS-CoV-2 Delta (B.1.617.2) variant of concern (VOC) and other VOCs are spreading in Europe. Micro-neutralisation assays with sera obtained after Comirnaty (BNT162b2, BioNTech/Pfizer) vaccination in 36 healthcare workers (31 female) demonstrated significant fold change reduction in neutralising titres compared with the original virus: Gamma (P.1) 2.3, Beta (B.1.351) 10.4, Delta 2.1 and 2.6. The reduction of the Alpha (B.1.1.7) variant was not significant. Despite being lower, remaining neutralisation capacity conferred by Comirnaty against Delta and other VOCs is probably protective.

Since its emergence, severe acute respiratory syndrome coronavirus-2 (SARS-CoV-2) has been responsible for more than 170 million cases and 3.5 million deaths. During December 2020 the Comirnaty (BNT162b2 mRNA, BioNTech-Pfizer, Mainz, Germany/New York, United States (US)) vaccine was approved by the US Food and Drug Administration and shown to be 95% efficacious in preventing symptomatic coronavirus disease 2019 (COVID-19) [1]. Clinical and real-world data demonstrated 95% effectiveness of the mRNA- based vaccine against the original SARS-CoV-2 and the Alpha (B.1.1.7) variant [1-3].

Since December 2020, several SARS-CoV-2 variants have emerged and were classified by the World Health Organization (WHO) as variants of concern (VOC): Alpha (Phylogenetic Assignment of Named Global Outbreak (Pango) lineage designation B.1.1.7), first detected in the United Kingdom (UK) [4], Beta (B.1.351) first documented in South Africa [5] and Gamma (P.1) initially detected in Brazil [6]. Most recently, in April 2021, the Delta (B.1.617.2) variant was identified in India and classified on May 11 as VOC due to its fast spread and potential immune escape [7]. Here, we describe the neutralising response of sera from healthcare workers without prior SARS-CoV-2 infection following a second vaccine dose against viral isolates of the Delta VOC, and compared it to the response against isolates of the original, the Alpha, Beta and Gamma VOCs.

## Whole genome sequencing

Following importation of SARS-CoV-2 variants by returning travellers, a national surveillance system has been set up in Israel, to sequence whole genomes of SARS-CoV-2-positive samples, to identify circulating and imported variants. In this analysis, we used the following isolates: the original (sub-lineage B.1 (hCoV-19/Israel/CVL-45526-ngs/2020), Alpha (hCoV-19/Israel/CVL-46879-ngs/2020), Beta (hCoV-19/Israel/CVL-2557-ngs/2020), and Delta sample 1 (S1, hCoV-19/Israel/CVL-12804/2021) and sample 2 (S2, hCoV-19/Israel/CVL-12806/2021). 

Libraries are prepared using COVID-seq library preparation kit, as per manufacturer’s instructions (Illumina, Cambridge, UK). Library validation and mean fragment size is determined by TapeStation 4200 via DNA HS D1000 kit (Agilent Technologies, Santa Clara, United States) and libraries are pooled, denatured and diluted to 10pM and sequenced on NovaSeq (Illumina). Sequences were mapped to the SARS-CoV-2 reference genomes (NC_045512.2) with Burrows-Wheeler aligner (BWA) mem [8]. Identification of the variants were done with Pangolin COVID-19 lineage assigner (https://pangolin.cog-uk.io/) and specific mutations were identified with a custom python code and Nextclade (https://clades.nextstrain.org/).

**Figure 1:**
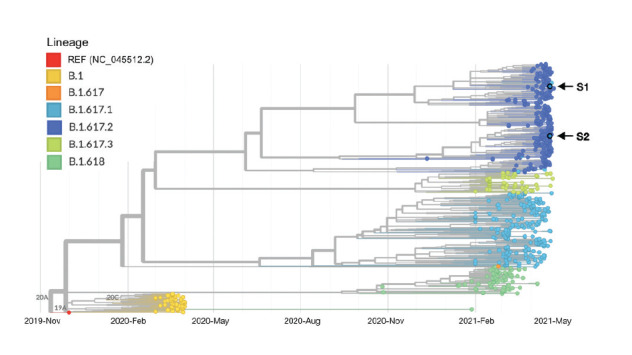
Phylogenetic tree of SARS-CoV-2 VOC Delta isolates originating in India

The two Delta VOC isolates, which differed by few mutations (S1. Amino acid substitutions of the Delta isolates), were further analysed together with additional global sequences downloaded from the Global initiative on sharing all influenza data (GISAID) hCoV-19 database identified as belonging to several main variants originating in India – Delta, Kappa (B.1.617.1), as well as the non-VOC B.1.617.3 and B.1.618, and their background lineage B.1 (S2. GISAID accession numbers of global variant sequences included in the phylogenetic tree). Phylogenetic trees were constructed using the Augur pipeline [9] (Figure 1).

SARS-CoV-2: severe acute respiratory syndrome coronavirus-2, VOC: variant of concern.

B.1.617 and B.1.618 (non-VOC) lineages, and their background lineage B.1 (original virus) were constructed using Nextstrain’s Augur pipeline and visualised with Auspice [9]. The two SARS-CoV-2 VOC Delta isolates, S1 and S2, marked with a black circle, are located in different branches of the B.1.617.2 lineage.

## Viral isolation of variants of concern 

All VOCs, were isolated from 300 µl of nasopharyngeal samples by incubation with confluent VERO-E6 cells for 1 hour at 33 °C followed by addition of 5 ml 2% Fetal bovine serum (FCS) Minimum Essential Medium (MEM)-Eagle medium. The Gamma isolate was kindly provided by the Tropical Medicine Institute, Sao Paulo University (Brazil) [6]. Confirmation of VOC identities was established by sequencing of all isolated variants.

## Ethical statement

The protocols (numbers: SMC-8008-20, SMC-7875-20) were approved by the Institutional review board of the Sheba Medical Center.

## Testing neutralisation capacity of variants of concern using SARS-CoV-2 micro-neutralisation assay

Neutralising antibodies against all VOCs were tested in serum samples obtained from healthy health care workers (HCW) of Sheba Medical Center 1 month following the receipt of the second Comirnaty vaccine dose. Because of limited availability of sera to test all isolates, samples from two different cohorts originating from the same study were tested. The first cohort consisted of 19 HCW (17 women and 2 men, median age 48, interquartile range (IQR) 18) and their sera were tested against the original B.1 virus and the two Delta VOC isolates. The second cohort was composed of 15 HCW (12 women and 3 men, median age 46, IQR 15) and their sera were tested against the original virus, as well as the Alpha, Beta and Gamma VOC.

Following titration of the original and VOCs, 100 median tissue culture infectious dose (TCID)50 of SARS-CoV-2 isolates were incubated with inactivated serum samples diluted 1:8 to 1:16,384 in 96 well plates for 60 min at 33 °C. Virus-serum mixtures were added to the VERO-E6 cells and incubated for 5 days at 33 °C after which Gentain violet staining (1%) was used to stain and fix the cell culture layer. Neutralising dilution of each serum sample was determined by identifying the well with the highest serum dilution without observable cytopathic effect. A dilution equal to 1:10 or above was considered neutralising [10].

**Figure 2:**
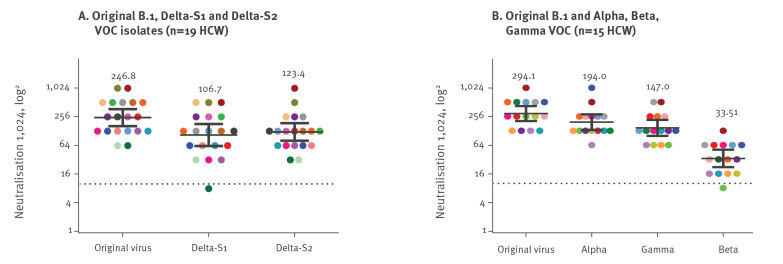
**Neutralisation titres of individuals vaccinated with a second dose of** **Comirnaty^a^** **against SARS-CoV-2 variants of concern**

Serum samples neutralised the original, Delta-S1 and Delta-S2 virus isolates with geometric mean titres (GMT) of 247, 107 and 123, respectively (Figure 2a), demonstrating a twofold reduction in neutralising titres compared with the original virus in vaccinated individuals. Statistical analysis was performed with the use of the Wilcoxon matched-pairs signed-rank test. The statistical significance of the differences between GMT in the original virus neutralisation assay and Delta-S1 and Delta-S2 neutralisation assays was p < 0.0001 for both isolates.

GMT: geometric mean titre; SARS-CoV-2; severe acute respiratory syndrome coronavirus-2, VOC: variant of concern.

^a^ BNT162b2 mRNA, BioNTech-Pfizer, Mainz, Germany/New York, United States (US).

Neutralisation levels and GMT against the original non-VOC B.1 SARS-CoV-2 virus, as well as Alpha, Beta, and Gamma SARS-CoV-2 VOCs are presented. The dotted black line indicates limit of positive neutralisation titres (cut off titre). The horizontal and 𝙸 bars indicate geometric mean titres and 95% confidence intervals, respectively.

**Figure 3:**
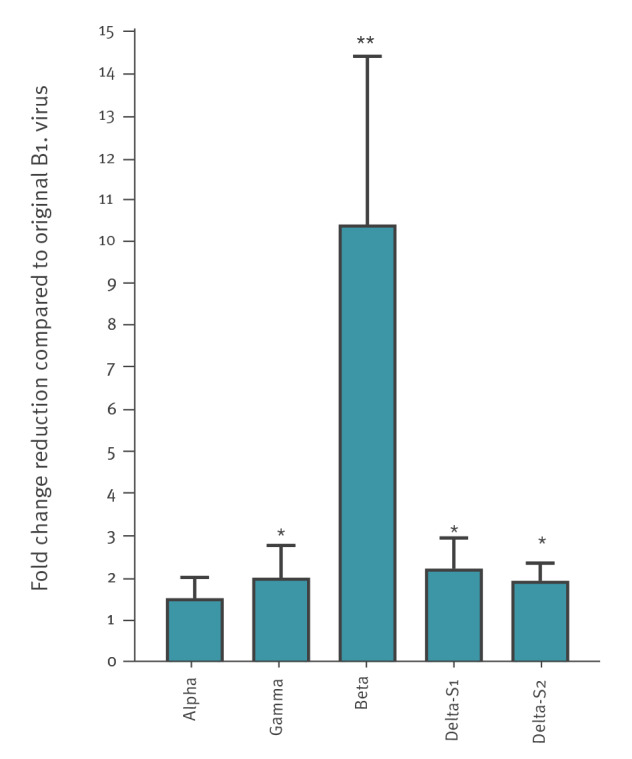
Fold change reduction compared to the original B.1 non-VOC SARS-CoV-2 in neutralising activity of VOCs

Neutralising titres against the original virus, and the Alpha, Gamma and Beta VOCs were GMT 294, 194, 147 and 33.5, respectively (Figure 2b). Overall, the following fold change reduction in neutralising titres were observed compared with the original virus: Alpha 1.7 (95% confidence interval (CI): 1.2–2.1), Gamma 2.3 (95% CI: 1.6–3), Beta 10.4 (95% CI: 6.4–14.4), Delta-S1 2.6 (95% CI: 1.8–3.5) and Delta-S2 2.1 (95% CI: 1.7–2.5) (Figure 3). Wilcoxon matched-pairs signed-rank test demonstrated significant differences in neutralising titres between the original virus and Beta (p < 0.0001), Gamma (p = 0.002) and both Delta VOC isolates (p < 0.0001) but not with the Alpha VOC.

CI: confidence interval; SARS-CoV-2; severe acute respiratory syndrome coronavirus-2, VOC: variant of concern.

The graph shows fold change reduction compared to the original B.1 non-VOC SARS-CoV-2 variants. The bars percent 95% CI. Wilcoxon matched-pairs signed-rank test demonstrated significant differences in neutralising titres between the original virus and the variants (p < 0.0001 ** and p = 0.002 *).

## Discussion and Conclusion

Available data suggest that vaccination with Comirnaty is effective against the Alpha, Beta and Gamma VOCs, albeit at different degrees [10-12]. The presence of mutations such as L452R and P681R in the SARS-CoV-2 spike protein’s receptor binding domain of the Delta variant, shown to be associated with high transmissibility [13] in addition to the fast spread and surge of severe cases of this variant in the Indian sub-continent in recent months, raised concern regarding the impact of this variant and its ability to evade SARS-CoV-2 vaccines [14]. We demonstrate here that neutralising levels against both Delta isolates were significantly reduced, although only by twofold compared with the original virus. Indeed, a recent study similarly showed that the Delta VOC is reduced by 2.5 folds compared to a Wuhan-related SARS-CoV-2 strain in individuals 4 to 14 days following the second dose of Comirnaty [15]. While only a 1.7-fold reduction was observed for the Alpha VOC compared with the original virus, our results show a 10-fold reduction in neutralising titres against the Beta VOC and twofold reduction against the Gamma VOC. Indeed, a recent study showed that neutralising titres against both the Beta and Gamma VOCs are reduced by eight to 12-fold and fourfold, respectively [11,16,17]. Interestingly, effectiveness of the Comirnaty vaccine was recently shown to be 75% against any documented infection with the Beta variant and 97.4% against severe, critical, or fatal disease [18]. Since reduction in neutralising levels against the Delta was significantly less than the Beta variant, these data suggest that Comirnaty vaccination is most probably, protective against the Delta VOC.

The data presented here contribute to the growing evidence of effectiveness of the mRNA-based Comirnaty vaccine against known VOCs and highlight the importance of vaccination specifically in areas with high proportion of VOC circulation Strengths of this study are the use of wild type isolated viruses and not mutated pseudo-viruses to evaluate the full neutralizing response and assessing neutralisation of two different and phylogenetically distinct Delta isolates. Limitations of the study include the small number of sera analysed, the strong over representation of women (31 of 36 participants) and the lack of T-cell response evaluation. In this study we assessed the neutralising capacity of sera from the vaccinated HCW against the Delta and not Kappa VOC. The Kappa VOC also originated in India where it has been circulating since October 2020, albeit Delta is the dominant sub-lineage circulating. 

Overall our results suggest that despite somewhat reduced neutralisation capacity, Comirnaty vaccination induces a substantial antibody response also for the Delta VOC. Further studies are necessary to confirm the vaccine effectiveness in broader population groups. 

## Supplementary Data

189000 Supplement
