# Supplementary material for: Neutralising capacity against Delta (B.1.617.2) and other variants of concern following Comirnaty (BNT162b2, BioNTech/Pfizer) vaccination in health care workers, Israel
Source: Euro Surveill. 2021 Jul 1;26(26):2100557. doi: 10.2807/1560-7917.ES.2021.26.26.2100557 (PMC8326656; doi:10.2807/1560-7917.ES.2021.26.26.2100557)
Supplement: Supplement [file 21-00557_MANDELBOIM-supplement.pdf]

Supplementary Material

This supplementary material is hosted by *Eurosurveillance* as supporting information alongside the article “Neutralising capacity against Delta (B.1.617.2) and other variants of concern following Comirnaty (BNT162b2, BioNTech/Pfizer) vaccination in health care workers, Israel” on behalf of the authors who remain responsible for the accuracy and appropriateness of the content. The same standards for ethics, copyright, attributions and permissions as for the article apply. Supplements are not edited by Eurosurveillance and the journal is not responsible for the maintenance of any links or email addresses provided therein.

Supplementary Table 1: Amino acid substitutions of the Delta isolates

Amino acid substitutions were identified in each sequence in comparison to SARS-CoV-2 reference sequence (REF_NC_045512.2).

**S1**	N:R385K, ORF7a:L116F, ORF1a:P309L,ORF1a:P1640L,ORF1a:H2092Y, ORF1a:A3209V,ORF1a:V3718A, ORF1b:G662S,ORF1b:H2285Y
**S2**	S:T95I, N:G215C, ORF3a:L85F, ORF7b:T40I,ORF1a:A1306S,ORF1a:P2046L, ORF1a:P2287S,ORF1a:V2930L,ORF1a:T3255I,ORF1a:T3646A, ORF1b:P314L,ORF1b:G662S,ORF1b:A1918V
**S1+S2**	M:I82T,N:D63G,N:R203M,N:D377Y, ORF1b:P314L,ORF1b:G662S, ORF1b:P1000L, ORF3a:S26L,ORF7a:V82A,ORF7a:T120I, S:T19R,S:R158G, S:L452R,S:T478K,S:D614G,S:P681R,S:D950N

Supplementary Table 2: GISAID accession numbers of global variant sequences included in the phylogenetic tree

**B.1**	hCoV-19/France/IDF_PSL_59/2020|EPI_ISL_1015006|2020-03-11<br>hCoV-19/France/IDF_PSL_47/2020|EPI_ISL_1015007|2020-03-20<br>hCoV-19/France/IDF_PSL_77/2020|EPI_ISL_1015008|2020-03-21<br>hCoV-19/France/IDF_PSL_71/2020|EPI_ISL_1015009|2020-03-23

	hCoV-19/France/IDF_PSL_58/2020|EPI_ISL_1015013|2020-03-19<br>hCoV-19/France/IDF_PSL_3/2020|EPI_ISL_1015023|2020-03-11<br>hCoV-19/France/IDF_PSL_160/2020|EPI_ISL_1015048|2020-03-06<br>hCoV-19/France/IDF_PSL_119/2020|EPI_ISL_1015258|2020-03-23<br>hCoV-19/Italy/FVG-ICGEB-S1/2020|EPI_ISL_417418|2020-03-01<br>hCoV-19/Italy/FVG-ICGEB-S5/2020|EPI_ISL_417419|2020-03-01<br>hCoV-19/Italy/FVG-ICGEB-S8/2020|EPI_ISL_417421|2020-03-01<br>hCoV-19/Georgia/Tb-1352/2020|EPI_ISL_420140|2020-03-28<br>hCoV-19/Slovenia/4584/2020|EPI_ISL_420294|2020-03-06<br>hCoV-19/USA/ID-UW-4486/2020|EPI_ISL_427172|2020-03-29<br>hCoV-19/Taiwan/TSGH-07/2020|EPI_ISL_427394|2020-03-22<br>hCoV-19/Taiwan/TSGH-12/2020|EPI_ISL_428229|2020-03-18<br>hCoV-19/Italy/FVG-ICGEB-S14/2020|EPI_ISL_428853|2020-03-30<br>hCoV-19/USA/CT-UW-4348/2020|EPI_ISL_430946|2020-03-30<br>hCoV-19/USA/ID-UW-4402/2020|EPI_ISL_430949|2020-03-29<br>hCoV-19/USA/OR-UW-4410/2020|EPI_ISL_430950|2020-03-29<br>hCoV-19/USA/WA-UW-4413/2020|EPI_ISL_430951|2020-03-31<br>hCoV-19/USA/ID-UW-6349/2020|EPI_ISL_430970|2020-04-01<br>hCoV-19/Taiwan/TSGH-18/2020|EPI_ISL_436103|2020-03-24<br>hCoV-19/Taiwan/TSGH-21/2020|EPI_ISL_436106|2020-03-30<br>hCoV-19/USA/un-UW-4089/2020|EPI_ISL_437818|2020-04-01<br>hCoV-19/USA/ID-UW-4308/2020|EPI_ISL_437828|2020-03-30<br>hCoV-19/Sweden/KI-CTMR-V300057201-8E/2020|EPI_ISL_444489|2020-03<br>hCoV-19/Chile/VS-ISPCH-4/2020|EPI_ISL_445287|2020-03-19<br>hCoV-19/Romania/Bucuresti-4322/2020|EPI_ISL_468136|2020-03-22<br>hCoV-19/Italy/APU-UniMI-847/2020|EPI_ISL_469022|2020-03-20<br>hCoV-19/USA/WA-UW-862/2020|EPI_ISL_476937|2020-03-14<br>hCoV-19/USA/WA-UW-1481/2020|EPI_ISL_477687|2020-03-18<br>hCoV-19/USA/ID-UW-3204/2020|EPI_ISL_490993|2020-03-26<br>hCoV-19/Taiwan/NCKUH-005/2020|EPI_ISL_493200|2020-03-18<br>hCoV-19/Italy/VEN-IZSV-9/2020|EPI_ISL_496482|2020-03-17<br>hCoV-19/USA/NY-UW-MAYO23/2020|EPI_ISL_500504|2020-03-18<br>hCoV-19/USA/TX-GCID-192000159/2020|EPI_ISL_501168|2020-03-30<br>hCoV-19/Italy/APU-UniMI-62PT/2020|EPI_ISL_525571|2020-03-28<br>hCoV-19/SaudiArabia/KAUST-MADINAH669/2020|EPI_ISL_677973|2020-04-01<br>hCoV-19/Italy/TUS-UniSi07/2020|EPI_ISL_803886|2020-03-18<br>hCoV-19/Italy/VEN-PD01/2020|EPI_ISL_856712|2020-03-24<br>hCoV-19/Italy/CAM_CRGS_2/2020|EPI_ISL_876042|2020-03-14<br>hCoV-19/Italy/CAM_CRGS_3/2020|EPI_ISL_876043|2020-03-14<br>hCoV-19/Italy/CAM-CRGS-36/2020|EPI_ISL_876977|2020-03-19<br>hCoV-19/Italy/CAM-CRGS-12/2020|EPI_ISL_876978|2020-03-15<br>hCoV-19/Italy/CAM-CRGS-29/2020|EPI_ISL_876993|2020-03-18<br>hCoV-19/Italy/CAM-CRGS-31/2020|EPI_ISL_876997|2020-03-19<br>hCoV-19/Italy/CAM-CRGS-37/2020|EPI_ISL_876999|2020-03-19<br>hCoV-19/Italy/CAM-CRGS-46/2020|EPI_ISL_877010|2020-03-25<br>hCoV-19/Italy/CAM-CRGS-57/2020|EPI_ISL_877011|2020-03-25<br>hCoV-19/Italy/CAM-CRGS-55/2020|EPI_ISL_918413|2020-03-19<br>hCoV-19/Italy/CAM-CRGS-63/2020|EPI_ISL_918416|2020-03-25
**B.1.617.1**	hCoV-19/India/MH-NCCS-87477/2021|EPI_ISL_1415316|2021-02-16<br>hCoV-19/India/MH-NCCS-87475/2021|EPI_ISL_1415323|2021-02-16<br>hCoV-19/England/CAMC-14A6515/2021|EPI_ISL_1504891|2021-03-28<br>hCoV-19/England/CAMC-14ABA6C/2021|EPI_ISL_1505110|2021-03-31<br>hCoV-19/USA/DE-DHSS-B1069933/2021|EPI_ISL_1511179|2021-03-29<br>hCoV-19/USA/GA-CDC-STM-000041012/2021|EPI_ISL_1512225|2021-03-21

	hCoV-19/England/CAMC-14B59F9/2021|EPI_ISL_1517951|2021-03-31<br>hCoV-19/Singapore/395/2021|EPI_ISL_1524796|2021-03-30<br>hCoV-19/India/MH-NCCS-9844/2021|EPI_ISL_1533801|2021-02-25<br>hCoV-19/England/CAMC-14C99D4/2021|EPI_ISL_1535006|2021-04-03<br>hCoV-19/England/CAMC-14CAB46/2021|EPI_ISL_1535035|2021-04-03<br>hCoV-19/England/MILK-14C78AC/2021|EPI_ISL_1536737|2021-04-03<br>hCoV-19/England/RAND-14C7156/2021|EPI_ISL_1537092|2021-04-03<br>hCoV-19/India/MH-NCCS-138900/2021|EPI_ISL_1544042|2021-02-26<br>hCoV-19/India/MH-NCCS-302014350031820/2021|EPI_ISL_1544065|2021-02-24<br>hCoV-19/India/MH-NCCS-RT231527/2021|EPI_ISL_1544068|2021-02-26<br>hCoV-19/Scotland/QEUH-14C89B7/2021|EPI_ISL_1544937|2021-04-02<br>hCoV-19/India/MH-NEERI-NGP-40449/2021|EPI_ISL_1547802|2021-03-10<br>hCoV-19/India/MH-NEERI-NGP-41290/2021|EPI_ISL_1547805|2021-03-13<br>hCoV-19/USA/CA-CDC-STM-000042578/2021|EPI_ISL_1576374|2021-03-24<br>hCoV-19/USA/PA-CDC-STM-000043645/2021|EPI_ISL_1581188|2021-03-24<br>hCoV-19/England/MILK-14D984D/2021|EPI_ISL_1584428|2021-04-05<br>hCoV-19/India/WB-1931300251031/2021|EPI_ISL_1589857|2021-03-25<br>hCoV-19/India/WB-1931500943932/2021|EPI_ISL_1589878|2021-03-12<br>hCoV-19/India/WB-1931300249539/2021|EPI_ISL_1589884|2021-03-22<br>hCoV-19/India/WB-1931300251000/2021|EPI_ISL_1589887|2021-03-25<br>hCoV-19/India/WB-1931300251003/2021|EPI_ISL_1589888|2021-03-25<br>hCoV-19/India/WB-1931300251005/2021|EPI_ISL_1589889|2021-03-25<br>hCoV-19/India/WB-1931300251518/2021|EPI_ISL_1589893|2021-03-26<br>hCoV-19/India/WB-1931300252032/2021|EPI_ISL_1589896|2021-03-27<br>hCoV-19/India/WB-1931500981090/2021|EPI_ISL_1589902|2021-03-22<br>hCoV-19/India/WB-1930400368956/2021|EPI_ISL_1589904|2021-02-26<br>hCoV-19/India/WB-1931500990949/2021|EPI_ISL_1589906|2021-03-24<br>hCoV-19/India/WB-1931501009007/2021|EPI_ISL_1589910|2021-03-29<br>hCoV-19/India/WB-1931501009410/2021|EPI_ISL_1589911|2021-03-29<br>hCoV-19/India/WB-1930300560861/2021|EPI_ISL_1589914|2021-03-09<br>hCoV-19/India/WB-1930300562863/2021|EPI_ISL_1589915|2021-03-10<br>hCoV-19/India/WB-1931500931775/2021|EPI_ISL_1589916|2021-03-09<br>hCoV-19/India/WB-1931500948337/2021|EPI_ISL_1589919|2021-03-13<br>hCoV-19/India/WB-1930300556491/2021|EPI_ISL_1589920|2021-03-07<br>hCoV-19/India/WB-1930300559242/2021|EPI_ISL_1589921|2021-03-08<br>hCoV-19/India/WB-1930300569917/2021|EPI_ISL_1589924|2021-03-13<br>hCoV-19/India/WB-1930300586246/2021|EPI_ISL_1589926|2021-03-19<br>hCoV-19/India/WB-1930300585938/2021|EPI_ISL_1589928|2021-03-19<br>hCoV-19/India/WB-1970400172259/2021|EPI_ISL_1589932|2021-03-14<br>hCoV-19/India/WB-1931500942112/2021|EPI_ISL_1589935|2021-03-12<br>hCoV-19/India/WB-1930400389876/2021|EPI_ISL_1589940|2021-03-11<br>hCoV-19/India/WB-1930400403451/2021|EPI_ISL_1589941|2021-03-19<br>hCoV-19/USA/GA-CDC-STM-000046368/2021|EPI_ISL_1593133|2021-03-31<br>hCoV-19/Wales/CAMC-14D5F6A/2021|EPI_ISL_1595472|2021-04-05<br>hCoV-19/Belgium/MBLG36792/2021|EPI_ISL_1599177|2021-03-25<br>hCoV-19/USA/WI-CDC-LC0035686/2021|EPI_ISL_1608983|2021-03-27<br>hCoV-19/USA/WA-UW-2021033003742/2021|EPI_ISL_1616618|2021-03-30<br>hCoV-19/USA/WA-UW-2021040107121/2021|EPI_ISL_1620998|2021-04-01<br>hCoV-19/USA/WA-UW-2021040100156/2021|EPI_ISL_1620999|2021-04-01<br>hCoV-19/England/RAND-14F1A67/2021|EPI_ISL_1631230|2021-04-09<br>hCoV-19/England/RAND-14F18D6/2021|EPI_ISL_1634737|2021-04-10<br>hCoV-19/Germany/BY-RKI-I-076082/2021|EPI_ISL_1641831|2021-04-06<br>hCoV-19/SouthKorea/KDCA2949/2021|EPI_ISL_1647348|2021-03-26<br>hCoV-19/SouthKorea/KDCA2950/2021|EPI_ISL_1647349|2021-04-05

	hCoV-19/SouthKorea/KDCA2951/2021|EPI_ISL_1647350|2021-04-06<br>hCoV-19/SouthKorea/KDCA2952/2021|EPI_ISL_1647351|2021-04-06<br>hCoV-19/SouthKorea/KDCA2953/2021|EPI_ISL_1647352|2021-04-06<br>hCoV-19/England/CAMC-14F8523/2021|EPI_ISL_1652951|2021-04-14<br>hCoV-19/England/CAMC-14F3C7E/2021|EPI_ISL_1653362|2021-04-09<br>hCoV-19/Switzerland/SO-ETHZ-560325/2021|EPI_ISL_1658295|2021-03-30<br>hCoV-19/Australia/NSW-R0186/2021|EPI_ISL_1660404|2021-04-07<br>hCoV-19/India/CT-ILSGS00361/2021|EPI_ISL_1662307|2021-02-23<br>hCoV-19/India/CT-ILSGS00371/2021|EPI_ISL_1662316|2021-02-23<br>hCoV-19/India/CT-ILSGS00373/2021|EPI_ISL_1662318|2021-02-25<br>hCoV-19/India/CT-ILSGS00378/2021|EPI_ISL_1662322|2021-02-18<br>hCoV-19/India/CT-ILSGS00482/2021|EPI_ISL_1662410|2021-02-15<br>hCoV-19/India/CT-ILSGS00494/2021|EPI_ISL_1662420|2021-02-22<br>hCoV-19/India/OR-ILSGS00645/2021|EPI_ISL_1662427|2021-03-25<br>hCoV-19/India/OR-ILSGS00649/2021|EPI_ISL_1662431|2021-03-25<br>hCoV-19/India/ILSGS00512/2021|EPI_ISL_1663243|2021-03-05<br>hCoV-19/India/ILSGS00522/2021|EPI_ISL_1663250|2021-03-06<br>hCoV-19/India/ILSGS00622/2021|EPI_ISL_1663315|2021-03-15<br>hCoV-19/India/ILSGS00623/2021|EPI_ISL_1663316|2021-03-15<br>hCoV-19/India/ILSGS00628/2021|EPI_ISL_1663320|2021-03-10<br>hCoV-19/India/ILSGS00753/2021|EPI_ISL_1663359|2021-03-22<br>hCoV-19/India/ILSGS00758/2021|EPI_ISL_1663362|2021-03-19<br>hCoV-19/India/ILSGS00762/2021|EPI_ISL_1663363|2021-03-07<br>hCoV-19/India/ILSGS00765/2021|EPI_ISL_1663365|2021-03-08<br>hCoV-19/India/ILSGS00769/2021|EPI_ISL_1663368|2021-03-07<br>hCoV-19/India/ILSGS00770/2021|EPI_ISL_1663369|2021-03-07<br>hCoV-19/India/ILSGS00783/2021|EPI_ISL_1663381|2021-03-02<br>hCoV-19/India/ILSGS00784/2021|EPI_ISL_1663382|2021-03-02<br>hCoV-19/India/ILSGS00789/2021|EPI_ISL_1663387|2021-03-03<br>hCoV-19/India/ILSGS00793/2021|EPI_ISL_1663391|2021-03-03<br>hCoV-19/India/ILSGS00797/2021|EPI_ISL_1663395|2021-03-04<br>hCoV-19/India/ILSGS00807/2021|EPI_ISL_1663406|2021-03-05<br>hCoV-19/India/ILSGS00808/2021|EPI_ISL_1663407|2021-03-06<br>hCoV-19/India/ILSGS00809/2021|EPI_ISL_1663408|2021-03-05<br>hCoV-19/India/ILSGS00811/2021|EPI_ISL_1663410|2021-03-06<br>hCoV-19/India/ILSGS00812/2021|EPI_ISL_1663411|2021-03-07<br>hCoV-19/India/ILSGS00822/2021|EPI_ISL_1663420|2021-03-07<br>hCoV-19/India/ILSGS00824/2021|EPI_ISL_1663422|2021-03-08<br>hCoV-19/India/ILSGS00825/2021|EPI_ISL_1663423|2021-03-08<br>hCoV-19/India/ILSGS00826/2021|EPI_ISL_1663424|2021-03-05<br>hCoV-19/India/ILSGS00832/2021|EPI_ISL_1663429|2021-03-05<br>hCoV-19/India/ILSGS00839/2021|EPI_ISL_1663434|2021-03-05<br>hCoV-19/India/ILSGS00850/2021|EPI_ISL_1663444|2021-03-05<br>hCoV-19/India/ILSGS00857/2021|EPI_ISL_1663448|2021-03-05<br>hCoV-19/India/ILSGS00867/2021|EPI_ISL_1663454|2021-03-06<br>hCoV-19/India/ILSGS00868/2021|EPI_ISL_1663455|2021-03-06<br>hCoV-19/India/ILSGS00871/2021|EPI_ISL_1663458|2021-03-05<br>hCoV-19/India/ILSGS00876/2021|EPI_ISL_1663461|2021-03-05<br>hCoV-19/India/ILSGS00878/2021|EPI_ISL_1663463|2021-03-06<br>hCoV-19/India/ILSGS00885/2021|EPI_ISL_1663469|2021-03-07<br>hCoV-19/India/ILSGS00887/2021|EPI_ISL_1663471|2021-03-07<br>hCoV-19/India/ILSGS00893/2021|EPI_ISL_1663476|2021-02-20<br>hCoV-19/India/ILSGS00910/2021|EPI_ISL_1663489|2021-04-02<br>hCoV-19/India/ILSGS00913/2021|EPI_ISL_1663492|2021-04-02

	hCoV-19/India/ILSGS00933/2021|EPI_ISL_1663509|2021-03-16<br>hCoV-19/India/ILSGS00945/2021|EPI_ISL_1663518|2021-03-19<br>hCoV-19/India/ILSGS00949/2021|EPI_ISL_1663522|2021-03-22<br>hCoV-19/India/ILSGS00957/2021|EPI_ISL_1663530|2021-03-26<br>hCoV-19/India/MH-ICMR-MGIMSF1/2021|EPI_ISL_1669766|2021-02<br>hCoV-19/India/MH-ICMR-MGIMSF5/2021|EPI_ISL_1669767|2021-02<br>hCoV-19/India/MH-ICMR-MGIMSF6/2021|EPI_ISL_1669768|2021-02<br>hCoV-19/India/MH-ICMR-MGIMSF7/2021|EPI_ISL_1669769|2021-02<br>hCoV-19/India/MH-ICMR-MGIMSF8/2021|EPI_ISL_1669770|2021-02<br>hCoV-19/India/MH-ICMR-2747200103301/2021|EPI_ISL_1669775|2021-02<br>hCoV-19/India/MH-ICMR-NGP173249/2021|EPI_ISL_1669777|2021-02<br>hCoV-19/India/MH-ICMR-NGP174677/2021|EPI_ISL_1669778|2021-02<br>hCoV-19/USA/WA-UW-2021041209221/2021|EPI_ISL_1670838|2021-04-12<br>hCoV-19/USA/CA-Stanford-15_S12/2021|EPI_ISL_1675224|2021-03-12<br>hCoV-19/USA/CA-Stanford-15_S27/2021|EPI_ISL_1675225|2021-03-16<br>hCoV-19/USA/CA-Stanford-19_S07/2021|EPI_ISL_1675226|2021-04-06<br>hCoV-19/USA/CA-Stanford-19_S21/2021|EPI_ISL_1675227|2021-04-07<br>hCoV-19/India/GJ-GBRC540a/2021|EPI_ISL_1677768|2021-04-01<br>hCoV-19/USA/FL-CDC-STM-000049137/2021|EPI_ISL_1679168|2021-04-01<br>hCoV-19/USA/WA-UW-2021040707686/2021|EPI_ISL_1680320|2021-04-07<br>hCoV-19/USA/WA-UW-2021040824300/2021|EPI_ISL_1680383|2021-04-08<br>hCoV-19/USA/WA-UW-2021040875818/2021|EPI_ISL_1680385|2021-04-08<br>hCoV-19/USA/WI-CDC-LC0041862/2021|EPI_ISL_1682101|2021-04-02<br>hCoV-19/England/CAMC-15027A7/2021|EPI_ISL_1697772|2021-04-12<br>hCoV-19/England/CAMC-1508E15/2021|EPI_ISL_1698270|2021-04-12<br>hCoV-19/England/CAMC-1508D54/2021|EPI_ISL_1698328|2021-04-14<br>hCoV-19/Wales/ALDP-14F4484/2021|EPI_ISL_1700508|2021-04-09<br>hCoV-19/Wales/ALDP-14F482E/2021|EPI_ISL_1700513|2021-04-09<br>hCoV-19/USA/CA-Stanford-15_S45/2021|EPI_ISL_1701681|2021-03-28<br>hCoV-19/Netherlands/NH-RIVM-26500/2021|EPI_ISL_1705973|2021-04-06<br>hCoV-19/Netherlands/NH-RIVM-26512/2021|EPI_ISL_1705985|2021-04-06<br>hCoV-19/Cambodia/381152/2021|EPI_ISL_1706599|2021-04-05<br>hCoV-19/Greece/181/2021|EPI_ISL_1716737|2021-04-06<br>hCoV-19/England/ALDP-150F6BC/2021|EPI_ISL_1718340|2021-04-15<br>hCoV-19/England/MILK-150FA47/2021|EPI_ISL_1718386|2021-04-14<br>hCoV-19/England/MILK-151258C/2021|EPI_ISL_1719083|2021-04-16<br>hCoV-19/England/MILK-1512D84/2021|EPI_ISL_1719097|2021-04-17<br>hCoV-19/Portugal/PT5880/2021|EPI_ISL_1739170|2021-04-09<br>hCoV-19/Portugal/PT5891/2021|EPI_ISL_1739180|2021-04-13<br>hCoV-19/England/ALDP-15146D2/2021|EPI_ISL_1741248|2021-04-16<br>hCoV-19/England/ALDP-15146E1/2021|EPI_ISL_1741251|2021-04-15<br>hCoV-19/England/MILK-1517684/2021|EPI_ISL_1741365|2021-04-18<br>hCoV-19/England/MILK-15176DF/2021|EPI_ISL_1741430|2021-04-18<br>hCoV-19/Scotland/QEUH-1506F50/2021|EPI_ISL_1742109|2021-04-14<br>hCoV-19/Scotland/CAMC-1502ADB/2021|EPI_ISL_1742203|2021-04-11<br>hCoV-19/Scotland/CAMC-1507BF1/2021|EPI_ISL_1742292|2021-04-13<br>hCoV-19/Belgium/UZA-UA-CV2066039473/2021|EPI_ISL_1745202|2021-03-29<br>hCoV-19/Italy/VEN-IZSVe-21RS970-9_VI/2021|EPI_ISL_1745236|2021-04-14<br>hCoV-19/England/MILK-151C9D1/2021|EPI_ISL_1758222|2021-04-05<br>hCoV-19/England/MILK-151C84D/2021|EPI_ISL_1758228|2021-04-06<br>hCoV-19/USA/WA-UW-2021041090429/2021|EPI_ISL_1785703|2021-04-10<br>hCoV-19/USA/WA-UW-2021041585567/2021|EPI_ISL_1785914|2021-04-15<br>hCoV-19/Malaysia/IMR_WC185230/2021|EPI_ISL_1787254|2021-04-10<br>hCoV-19/USA/WA-UW-2021040809736/2021|EPI_ISL_1789270|2021-04-08

	hCoV-19/USA/WA-UW-2021041268513/2021|EPI_ISL_1789413|2021-04-12<br>hCoV-19/USA/WA-UW-2021041244403/2021|EPI_ISL_1789415|2021-04-12<br>hCoV-19/England/MILK-151F58F/2021|EPI_ISL_1790246|2021-04-09<br>hCoV-19/England/MILK-1523236/2021|EPI_ISL_1790678|2021-04-20<br>hCoV-19/Switzerland/ZH-UZH-IMV-3ba43fa0/2021|EPI_ISL_1791067|2021-03-29<br>hCoV-19/Netherlands/NB-RIVM-27595/2021|EPI_ISL_1792908|2021-04-15<br>hCoV-19/USA/WI-WSLH-213515/2021|EPI_ISL_1794390|2021-03-31<br>hCoV-19/USA/FL-CDC-STM-000053252/2021|EPI_ISL_1796460|2021-04-09<br>hCoV-19/USA/WI-CDC-LC0046190/2021|EPI_ISL_1800009|2021-04-07<br>hCoV-19/USA/VA-CDC-LC0047059/2021|EPI_ISL_1800850|2021-04-14<br>hCoV-19/USA/IL-CDC-STM-000053846/2021|EPI_ISL_1804291|2021-04-11<br>hCoV-19/USA/CA-CDC-STM-000054394/2021|EPI_ISL_1804713|2021-04-11<br>hCoV-19/England/MILK-1528617/2021|EPI_ISL_1806481|2021-04-21<br>hCoV-19/England/ALDP-152BE1C/2021|EPI_ISL_1806530|2021-04-22<br>hCoV-19/England/ALDP-152C974/2021|EPI_ISL_1806669|2021-04-21
**B.1.617.2**	hCoV-19/USA/MA-CDC-STM-000044850/2021|EPI_ISL_1592421|2021-03-28<br>hCoV-19/England/CAMC-14E338B/2021|EPI_ISL_1594728|2021-04-06<br>hCoV-19/England/CAMC-14E2F97/2021|EPI_ISL_1594918|2021-04-06<br>hCoV-19/USA/NJ-CDC-LC0038223/2021|EPI_ISL_1611370|2021-03-31<br>hCoV-19/USA/IN-CDC-STM-000045992/2021|EPI_ISL_1615349|2021-03-29<br>hCoV-19/Australia/NSW4469/2021|EPI_ISL_1615596|2021-04-13<br>hCoV-19/England/CAMC-14E7B61/2021|EPI_ISL_1615751|2021-04-08<br>hCoV-19/England/CAMC-14E7A0A/2021|EPI_ISL_1615877|2021-04-08<br>hCoV-19/USA/WA-UW-2021040308606/2021|EPI_ISL_1620980|2021-04-03<br>hCoV-19/England/ALDP-14EDD1A/2021|EPI_ISL_1631042|2021-04-10<br>hCoV-19/England/RAND-14F1AD0/2021|EPI_ISL_1631164|2021-04-09<br>hCoV-19/England/CAMC-14E7563/2021|EPI_ISL_1631219|2021-04-07<br>hCoV-19/England/MILK-14BF397/2021|EPI_ISL_1631645|2021-04-03<br>hCoV-19/England/RAND-14DD366/2021|EPI_ISL_1631836|2021-04-06<br>hCoV-19/England/RAND-14E21BF/2021|EPI_ISL_1632122|2021-04-07<br>hCoV-19/England/RAND-14E1D70/2021|EPI_ISL_1632256|2021-04-07<br>hCoV-19/Australia/NSW4474/2021|EPI_ISL_1633348|2021-04-16<br>hCoV-19/Australia/NSW4485/2021|EPI_ISL_1633353|2021-04-16<br>hCoV-19/England/MILK-14F617C/2021|EPI_ISL_1634507|2021-04-11<br>hCoV-19/England/QEUH-14E86C9/2021|EPI_ISL_1634920|2021-04-07<br>hCoV-19/Scotland/QEUH-14E2498/2021|EPI_ISL_1635330|2021-04-08<br>hCoV-19/England/ALDP-14FC413/2021|EPI_ISL_1652724|2021-04-10<br>hCoV-19/England/ALDP-14FCF9A/2021|EPI_ISL_1652738|2021-04-12<br>hCoV-19/England/ALDP-14FCFD6/2021|EPI_ISL_1652763|2021-04-12<br>hCoV-19/England/ALDP-14FC659/2021|EPI_ISL_1652890|2021-04-10<br>hCoV-19/England/RAND-14FD37F/2021|EPI_ISL_1652897|2021-04-12<br>hCoV-19/England/CAMC-14F84CC/2021|EPI_ISL_1652981|2021-04-11<br>hCoV-19/England/CAMC-14F86F3/2021|EPI_ISL_1652993|2021-04-13<br>hCoV-19/England/CAMC-14E66AD/2021|EPI_ISL_1653090|2021-04-06<br>hCoV-19/England/ALDP-14F8A06/2021|EPI_ISL_1653187|2021-04-11<br>hCoV-19/England/CAMC-14E69A4/2021|EPI_ISL_1653197|2021-04-06<br>hCoV-19/Wales/CAMC-14E8016/2021|EPI_ISL_1653755|2021-04-08<br>hCoV-19/India/OR-ILSGS00333/2021|EPI_ISL_1662284|2021-03-06<br>hCoV-19/India/CT-ILSGS00342/2021|EPI_ISL_1662291|2021-03-14<br>hCoV-19/India/OR-ILSGS00682/2021|EPI_ISL_1662451|2021-03-18<br>hCoV-19/India/ILSGS00519/2021|EPI_ISL_1663247|2021-03-08<br>hCoV-19/India/ILSGS00614/2021|EPI_ISL_1663307|2021-03-15<br>hCoV-19/India/ILSGS00615/2021|EPI_ISL_1663308|2021-03-15<br>hCoV-19/India/ILSGS00766/2021|EPI_ISL_1663366|2021-03-08

	hCoV-19/India/ILSGS00767/2021|EPI_ISL_1663367|2021-03-08
	hCoV-19/India/ILSGS00912/2021|EPI_ISL_1663491|2021-04-02
	hCoV-19/India/ILSGS00922/2021|EPI_ISL_1663498|2021-03-24
	hCoV-19/India/ILSGS00925/2021|EPI_ISL_1663501|2021-03-28
	hCoV-19/India/ILSGS00926/2021|EPI_ISL_1663502|2021-03-28
	hCoV-19/India/ILSGS00931/2021|EPI_ISL_1663507|2021-03-16
	hCoV-19/India/ILSGS00941/2020|EPI_ISL_1663516|2020-12-12
	hCoV-19/India/ILSGS00950/2021|EPI_ISL_1663523|2021-03-20
	hCoV-19/India/ILSGS00956/2021|EPI_ISL_1663529|2021-03-25
	hCoV-19/India/ILSGS00961/2021|EPI_ISL_1663534|2021-03-28
	hCoV-19/India/ILSGS00969/2021|EPI_ISL_1663541|2021-03-22
	hCoV-19/India/ILSGS00971/2021|EPI_ISL_1663543|2021-03-19
	hCoV-19/India/ILSGS00977/2021|EPI_ISL_1663548|2021-02-19
	hCoV-19/India/ILSGS00978/2021|EPI_ISL_1663549|2021-02-19
	hCoV-19/India/ILSGS00983/2021|EPI_ISL_1663552|2021-03-25
	hCoV-19/India/ILSGS00984/2021|EPI_ISL_1663553|2021-03-25
	hCoV-19/India/ILSGS00985/2021|EPI_ISL_1663554|2021-03-25
	hCoV-19/India/ILSGS00986/2021|EPI_ISL_1663555|2021-03-25
	hCoV-19/India/ILSGS00987/2021|EPI_ISL_1663557|2021-03-25
	hCoV-19/India/ILSGS00988/2021|EPI_ISL_1663558|2021-03-25
	hCoV-19/India/ILSGS00991/2021|EPI_ISL_1663561|2021-03-25
	hCoV-19/India/ILSGS00992/2021|EPI_ISL_1663562|2021-03-25
	hCoV-19/India/ILSGS00993/2021|EPI_ISL_1663563|2021-03-25
	hCoV-19/India/ILSGS00996/2021|EPI_ISL_1663564|2021-03-29
	hCoV-19/USA/WA-UW-2021041002621/2021|EPI_ISL_1670827|2021-04-10
	hCoV-19/England/CAMC-14E66BC/2021|EPI_ISL_1673061|2021-04-06
	hCoV-19/Scotland/CAMC-14E685C/2021|EPI_ISL_1673100|2021-04-04
	hCoV-19/USA/NY-CDC-2-4177223/2021|EPI_ISL_1678079|2021-03-16
	hCoV-19/USA/IN-CDC-STM-000049219/2021|EPI_ISL_1679967|2021-04-01
	hCoV-19/USA/IN-CDC-STM-000049216/2021|EPI_ISL_1680048|2021-04-01
	hCoV-19/USA/NC-CDC-LC0043123/2021|EPI_ISL_1683986|2021-04-09
	hCoV-19/USA/NV-CDC-LC0044049/2021|EPI_ISL_1684717|2021-04-05
	hCoV-19/England/CAMC-1502B8D/2021|EPI_ISL_1697963|2021-04-12
	hCoV-19/England/CAMC-1502BD8/2021|EPI_ISL_1697964|2021-04-13
	hCoV-19/England/MILK-150A709/2021|EPI_ISL_1697977|2021-04-14
	hCoV-19/England/MILK-1509FF3/2021|EPI_ISL_1698144|2021-04-15
	hCoV-19/England/CAMC-1508B87/2021|EPI_ISL_1698273|2021-04-14
	hCoV-19/England/CAMC-1508EBB/2021|EPI_ISL_1698283|2021-04-13
	hCoV-19/England/ALDP-150864A/2021|EPI_ISL_1698325|2021-04-14
	hCoV-19/England/CAMC-1508DDC/2021|EPI_ISL_1698361|2021-04-13
	hCoV-19/England/CAMC-1508D72/2021|EPI_ISL_1698410|2021-04-13
	hCoV-19/England/ALDP-15089D8/2021|EPI_ISL_1698416|2021-04-14
	hCoV-19/England/CAMC-1507C85/2021|EPI_ISL_1698429|2021-04-14
	hCoV-19/England/CAMC-150904B/2021|EPI_ISL_1698436|2021-04-17
	hCoV-19/England/ALDP-1507FD7/2021|EPI_ISL_1698440|2021-04-15
	hCoV-19/England/ALDP-14FB55D/2021|EPI_ISL_1699101|2021-04-12
	hCoV-19/England/MILK-15060E4/2021|EPI_ISL_1699753|2021-04-13
	hCoV-19/England/MILK-1506406/2021|EPI_ISL_1699770|2021-04-14
	hCoV-19/England/MILK-14F524D/2021|EPI_ISL_1699979|2021-04-11
	hCoV-19/England/ALDP-14F228C/2021|EPI_ISL_1700236|2021-04-11
	hCoV-19/England/RAND-14F1DE6/2021|EPI_ISL_1700313|2021-04-11
	hCoV-19/USA/OK-CDC-2-4195490/2021|EPI_ISL_1711803|2021-04-05
	hCoV-19/USA/WA-UW-2021040810481/2021|EPI_ISL_1715449|2021-04-08
	hCoV-19/England/MILK-1514766/2021|EPI_ISL_1718344|2021-04-17

	hCoV-19/England/ALDP-1513697/2021|EPI_ISL_1718501|2021-04-16<br>hCoV-19/England/ALDP-150F84D/2021|EPI_ISL_1718588|2021-04-15<br>hCoV-19/England/ALDP-151837D/2021|EPI_ISL_1718630|2021-04-18<br>hCoV-19/England/ALDP-1511F16/2021|EPI_ISL_1718683|2021-04-16<br>hCoV-19/England/MILK-15152A0/2021|EPI_ISL_1718691|2021-04-16<br>hCoV-19/England/ALDP-150EDE5/2021|EPI_ISL_1718721|2021-04-15<br>hCoV-19/England/ALDP-151851D/2021|EPI_ISL_1718882|2021-04-17<br>hCoV-19/England/MILK-151413B/2021|EPI_ISL_1718934|2021-04-17<br>hCoV-19/England/MILK-151407A/2021|EPI_ISL_1718935|2021-04-17<br>hCoV-19/England/MILK-1512443/2021|EPI_ISL_1718951|2021-04-17<br>hCoV-19/England/CAMC-1510A43/2021|EPI_ISL_1718959|2021-04-16<br>hCoV-19/England/MILK-15128CF/2021|EPI_ISL_1718987|2021-04-17<br>hCoV-19/England/CAMC-1510B6E/2021|EPI_ISL_1718989|2021-04-16<br>hCoV-19/England/MILK-1513B89/2021|EPI_ISL_1719015|2021-04-16<br>hCoV-19/England/CAMC-1510A07/2021|EPI_ISL_1719027|2021-04-16<br>hCoV-19/England/CAMC-15109FB/2021|EPI_ISL_1719042|2021-04-16<br>hCoV-19/England/CAMC-1510A8F/2021|EPI_ISL_1719134|2021-04-16<br>hCoV-19/England/CAMC-1510AF8/2021|EPI_ISL_1719142|2021-04-16<br>hCoV-19/England/MILK-15140E3/2021|EPI_ISL_1719162|2021-04-17<br>hCoV-19/England/CAMC-150D690/2021|EPI_ISL_1719584|2021-04-14<br>hCoV-19/England/ALDP-150DBDD/2021|EPI_ISL_1719616|2021-04-15<br>hCoV-19/England/CAMC-150D241/2021|EPI_ISL_1719618|2021-04-14<br>hCoV-19/England/QEUH-150C51C/2021|EPI_ISL_1719728|2021-04-17<br>hCoV-19/England/CAMC-1502B32/2021|EPI_ISL_1719736|2021-04-12<br>hCoV-19/Germany/BY-RKI-I-085003/2021|EPI_ISL_1722241|2021-04-09<br>hCoV-19/Germany/NW-RKI-I-093994/2021|EPI_ISL_1728331|2021-04-20<br>hCoV-19/Wales/PHWC-PYD79R/2021|EPI_ISL_1730635|2021-04-14<br>hCoV-19/England/PHWC-PYD9D5/2021|EPI_ISL_1730650|2021-04-15<br>hCoV-19/USA/IL-CDC-STM-000051595/2021|EPI_ISL_1735500|2021-04-06<br>hCoV-19/England/CAMC-151FDF0/2021|EPI_ISL_1740580|2021-04-22<br>hCoV-19/England/RAND-1520530/2021|EPI_ISL_1740633|2021-04-19<br>hCoV-19/England/ALDP-15202B1/2021|EPI_ISL_1740654|2021-04-19<br>hCoV-19/England/RAND-1520785/2021|EPI_ISL_1740691|2021-04-19<br>hCoV-19/England/QEUH-151F8A4/2021|EPI_ISL_1740738|2021-04-20<br>hCoV-19/England/ALDP-151FF27/2021|EPI_ISL_1740750|2021-04-20<br>hCoV-19/England/RAND-152054F/2021|EPI_ISL_1740754|2021-04-19<br>hCoV-19/England/ALDP-152007B/2021|EPI_ISL_1740763|2021-04-20<br>hCoV-19/England/RAND-1520688/2021|EPI_ISL_1740766|2021-04-19<br>hCoV-19/England/ALDP-152022A/2021|EPI_ISL_1740778|2021-04-19<br>hCoV-19/England/CAMC-151FE0C/2021|EPI_ISL_1740781|2021-04-22<br>hCoV-19/England/MILK-1517FA7/2021|EPI_ISL_1740858|2021-04-18<br>hCoV-19/England/MILK-1517CBF/2021|EPI_ISL_1740882|2021-04-19<br>hCoV-19/England/QEUH-150DFE0/2021|EPI_ISL_1741081|2021-04-15<br>hCoV-19/England/QEUH-150E0A0/2021|EPI_ISL_1741129|2021-04-15<br>hCoV-19/England/ALDP-151462D/2021|EPI_ISL_1741186|2021-04-15<br>hCoV-19/England/QEUH-150DDD7/2021|EPI_ISL_1741259|2021-04-15<br>hCoV-19/Scotland/QEUH-150AE04/2021|EPI_ISL_1741651|2021-04-16<br>hCoV-19/Scotland/QEUH-150B665/2021|EPI_ISL_1741677|2021-04-14<br>hCoV-19/Scotland/QEUH-15001AB/2021|EPI_ISL_1741838|2021-04-13<br>hCoV-19/Belgium/UZA-UA-CV2115112682/2021|EPI_ISL_1745199|2021-04-21<br>hCoV-19/Singapore/658/2021|EPI_ISL_1752677|2021-04-19<br>hCoV-19/England/MILK-151C92C/2021|EPI_ISL_1758205|2021-04-06<br>hCoV-19/England/MILK-151CC14/2021|EPI_ISL_1758283|2021-04-06<br>hCoV-19/England/ALDP-151A706/2021|EPI_ISL_1758301|2021-04-19

	hCoV-19/England/ALDP-151A618/2021|EPI_ISL_1758376|2021-04-18<br>hCoV-19/England/CAMC-151AA2B/2021|EPI_ISL_1758509|2021-04-19<br>hCoV-19/England/CAMC-151AAD0/2021|EPI_ISL_1758558|2021-04-17<br>hCoV-19/England/CAMC-151AAA3/2021|EPI_ISL_1758645|2021-04-14<br>hCoV-19/Scotland/QEUH-151114D/2021|EPI_ISL_1758819|2021-04-17<br>hCoV-19/Scotland/QEUH-150DF1D/2021|EPI_ISL_1758829|2021-04-16<br>hCoV-19/England/ALDP-1519C9F/2021|EPI_ISL_1758890|2021-04-19<br>hCoV-19/England/MILK-1516ED8/2021|EPI_ISL_1758919|2021-04-19<br>hCoV-19/England/RAND-1515DDC/2021|EPI_ISL_1758982|2021-04-16<br>hCoV-19/England/ALDP-151B94B/2021|EPI_ISL_1759190|2021-04-18<br>hCoV-19/England/MILK-151BA39/2021|EPI_ISL_1759218|2021-04-06<br>hCoV-19/England/ALDP-151B92D/2021|EPI_ISL_1759274|2021-04-18<br>hCoV-19/England/MILK-151C35B/2021|EPI_ISL_1759278|2021-04-19<br>hCoV-19/England/MILK-151B486/2021|EPI_ISL_1759335|2021-04-19<br>hCoV-19/England/MILK-151C5CE/2021|EPI_ISL_1759847|2021-04-19<br>hCoV-19/USA/WA-UW-2021041375198/2021|EPI_ISL_1785682|2021-04-13<br>hCoV-19/USA/WA-UW-2021041244547/2021|EPI_ISL_1789403|2021-04-12<br>hCoV-19/USA/WA-UW-2021041231568/2021|EPI_ISL_1789408|2021-04-12<br>hCoV-19/USA/WA-UW-2021041224321/2021|EPI_ISL_1789424|2021-04-12<br>hCoV-19/USA/WA-UW-2021041275729/2021|EPI_ISL_1789426|2021-04-12<br>hCoV-19/England/RAND-151EC4F/2021|EPI_ISL_1790323|2021-04-18<br>hCoV-19/England/QEUH-151CFDF/2021|EPI_ISL_1790373|2021-04-19<br>hCoV-19/England/RAND-151ED2E/2021|EPI_ISL_1790415|2021-04-17<br>hCoV-19/England/ALDP-1522EAC/2021|EPI_ISL_1790461|2021-04-19<br>hCoV-19/England/MILK-1523843/2021|EPI_ISL_1790473|2021-04-20<br>hCoV-19/England/MILK-1523A5C/2021|EPI_ISL_1790475|2021-04-16<br>hCoV-19/England/MILK-1523CFC/2021|EPI_ISL_1790529|2021-04-20<br>hCoV-19/England/ALDP-1522D45/2021|EPI_ISL_1790538|2021-04-19<br>hCoV-19/England/ALDP-1522DCD/2021|EPI_ISL_1790547|2021-04-19<br>hCoV-19/England/MILK-1523667/2021|EPI_ISL_1790590|2021-04-20<br>hCoV-19/England/ALDP-1522CFD/2021|EPI_ISL_1790596|2021-04-19<br>hCoV-19/England/MILK-1523193/2021|EPI_ISL_1790610|2021-04-20<br>hCoV-19/England/MILK-15235B5/2021|EPI_ISL_1790613|2021-04-20<br>hCoV-19/England/MILK-1523CCF/2021|EPI_ISL_1790679|2021-04-16<br>hCoV-19/England/MILK-1526770/2021|EPI_ISL_1790728|2021-04-21<br>hCoV-19/England/MILK-15244B7/2021|EPI_ISL_1790753|2021-04-19<br>hCoV-19/England/MILK-152462A/2021|EPI_ISL_1790800|2021-04-19<br>hCoV-19/England/MILK-15248BB/2021|EPI_ISL_1790817|2021-04-18<br>hCoV-19/England/MILK-1524806/2021|EPI_ISL_1790848|2021-04-20<br>hCoV-19/England/MILK-15268F5/2021|EPI_ISL_1790883|2021-04-19<br>hCoV-19/England/MILK-15248CA/2021|EPI_ISL_1790888|2021-04-20<br>hCoV-19/England/MILK-152444E/2021|EPI_ISL_1790893|2021-04-17<br>hCoV-19/England/MILK-1524D16/2021|EPI_ISL_1790905|2021-04-21<br>hCoV-19/England/MILK-1526901/2021|EPI_ISL_1790928|2021-04-21<br>hCoV-19/England/MILK-1524781/2021|EPI_ISL_1790940|2021-04-17<br>hCoV-19/England/ALDP-15253C8/2021|EPI_ISL_1790990|2021-04-20<br>hCoV-19/England/MILK-15267AD/2021|EPI_ISL_1790998|2021-04-17<br>hCoV-19/Switzerland/AG-UZH-IMV-3ba47ad1/2021|EPI_ISL_1791066|2021-04-24<br>hCoV-19/Netherlands/NH-RIVM-27142/2021|EPI_ISL_1792906|2021-04-13<br>hCoV-19/Netherlands/NB-RIVM-27390/2021|EPI_ISL_1792907|2021-04-15<br>hCoV-19/USA/GA-CDC-STM-000052719/2021|EPI_ISL_1797026|2021-04-09<br>hCoV-19/USA/TN-CDC-STM-000052160/2021|EPI_ISL_1797206|2021-04-08<br>hCoV-19/USA/CA-CDC-LC0044861/2021|EPI_ISL_1799003|2021-04-12<br>hCoV-19/USA/CA-CDC-LC0045319/2021|EPI_ISL_1799336|2021-04-11

	hCoV-19/USA/NC-CDC-LC0047119/2021|EPI_ISL_1800981|2021-04-16<br>hCoV-19/USA/IL-CDC-LC0049072/2021|EPI_ISL_1802476|2021-04-15<br>hCoV-19/USA/IL-CDC-LC0049345/2021|EPI_ISL_1802644|2021-04-17<br>hCoV-19/USA/IL-CDC-STM-000055027/2021|EPI_ISL_1803921|2021-04-13<br>hCoV-19/USA/CA-CDC-STM-000055247/2021|EPI_ISL_1804135|2021-04-12<br>hCoV-19/England/ALDP-15273A8/2021|EPI_ISL_1805965|2021-04-21<br>hCoV-19/England/MILK-1528626/2021|EPI_ISL_1806406|2021-04-22<br>hCoV-19/England/MILK-1528538/2021|EPI_ISL_1806433|2021-04-21<br>hCoV-19/England/ALDP-152C437/2021|EPI_ISL_1806511|2021-04-22<br>hCoV-19/England/ALDP-152CACC/2021|EPI_ISL_1806517|2021-04-22<br>hCoV-19/England/ALDP-152BB42/2021|EPI_ISL_1806521|2021-04-21<br>hCoV-19/England/ALDP-152CF27/2021|EPI_ISL_1806561|2021-04-22<br>hCoV-19/England/MILK-152C798/2021|EPI_ISL_1806601|2021-04-21<br>hCoV-19/England/ALDP-152BCA9/2021|EPI_ISL_1806670|2021-04-21<br>hCoV-19/England/MILK-152C82C/2021|EPI_ISL_1806709|2021-04-22<br>hCoV-19/England/MILK-152C65F/2021|EPI_ISL_1806761|2021-04-21<br>hCoV-19/England/MILK-152C4FB/2021|EPI_ISL_1806767|2021-04-22<br>hCoV-19/England/ALDP-152C40A/2021|EPI_ISL_1806837|2021-04-22<br>hCoV-19/England/ALDP-152C042/2021|EPI_ISL_1806852|2021-04-22<br>hCoV-19/Scotland/QEUH-15227ED/2021|EPI_ISL_1806892|2021-04-20<br>hCoV-19/Scotland/MILK-1524833/2021|EPI_ISL_1806897|2021-04-20<br>hCoV-19/Scotland/MILK-152488E/2021|EPI_ISL_1806899|2021-04-20<br>hCoV-19/NorthernIreland/RAND-151F710/2021|EPI_ISL_1806953|2021-04-20<br>hCoV-19/Sweden/1067735009/2021|EPI_ISL_1808686|2021-04-01<br>hCoV-19/Switzerland/GE-33896105/2021|EPI_ISL_1811202|2021-04-17<br>hCoV-19/NewZealand/21MV0340/2021|EPI_ISL_1621314|2021-04-09<br>hCoV-19/USA/WA-UW-2021040507831/2021|EPI_ISL_1628185|2021-04-05<br>hCoV-19/England/MILK-14DDCD4/2021|EPI_ISL_1631923|2021-04-06<br>hCoV-19/England/CAMC-14DEBF4/2021|EPI_ISL_1632316|2021-04-06<br>hCoV-19/England/CAMC-14DECF1/2021|EPI_ISL_1632335|2021-04-06<br>hCoV-19/England/CAMC-14DEBA9/2021|EPI_ISL_1632426|2021-04-06<br>hCoV-19/England/QEUH-14E884B/2021|EPI_ISL_1634973|2021-04-07<br>hCoV-19/England/CAMC-14DEE28/2021|EPI_ISL_1635371|2021-04-07<br>hCoV-19/Scotland/CAMC-14DEB6D/2021|EPI_ISL_1635379|2021-04-07<br>hCoV-19/England/RAND-14FD46D/2021|EPI_ISL_1652717|2021-04-12<br>hCoV-19/England/CAMC-15034AF/2021|EPI_ISL_1697491|2021-04-11<br>hCoV-19/England/CAMC-1502956/2021|EPI_ISL_1697919|2021-04-11<br>hCoV-19/England/MILK-150939D/2021|EPI_ISL_1698106|2021-04-15<br>hCoV-19/England/MILK-15056B6/2021|EPI_ISL_1699676|2021-04-14<br>hCoV-19/England/ALDP-14F4008/2021|EPI_ISL_1700038|2021-04-11<br>hCoV-19/USA/WA-UW-2021041370801/2021|EPI_ISL_1715530|2021-04-13<br>hCoV-19/Greece/180491/2021|EPI_ISL_1716736|2021-03-23<br>hCoV-19/England/ALDP-1513961/2021|EPI_ISL_1718381|2021-04-16<br>hCoV-19/England/ALDP-150F79B/2021|EPI_ISL_1718598|2021-04-15<br>hCoV-19/England/RAND-1514D91/2021|EPI_ISL_1718700|2021-04-15<br>hCoV-19/England/CAMC-15107C4/2021|EPI_ISL_1718942|2021-04-16<br>hCoV-19/England/MILK-1512319/2021|EPI_ISL_1719212|2021-04-17<br>hCoV-19/England/QEUH-150C52B/2021|EPI_ISL_1719573|2021-04-17<br>hCoV-19/England/PHWC-PYRYFW/2021|EPI_ISL_1730804|2021-04-15<br>hCoV-19/England/ALDP-151FEA2/2021|EPI_ISL_1740707|2021-04-19<br>hCoV-19/England/QEUH-150E2E6/2021|EPI_ISL_1741213|2021-04-15<br>hCoV-19/Scotland/QEUH-1500A64/2021|EPI_ISL_1741909|2021-04-13<br>hCoV-19/England/ALDP-1526A4A/2021|EPI_ISL_1790745|2021-04-20<br>hCoV-19/England/MILK-152461B/2021|EPI_ISL_1790832|2021-04-17

	hCoV-19/England/MILK-1524763/2021|EPI_ISL_1791005|2021-04-19<br>hCoV-19/Aruba/AW-RIVM-27687/2021|EPI_ISL_1792922|2021-04-16<br>hCoV-19/USA/NY-CDC-LC0046732/2021|EPI_ISL_1800829|2021-04-15<br>hCoV-19/USA/MD-CDC-LC0047180/2021|EPI_ISL_1801017|2021-04-16<br>hCoV-19/USA/NC-CDC-STM-000053861/2021|EPI_ISL_1804282|2021-04-11<br>hCoV-19/England/QEUH-1528DF4/2021|EPI_ISL_1806236|2021-04-21<br>hCoV-19/England/MILK-152877E/2021|EPI_ISL_1806342|2021-04-22<br>hCoV-19/England/QEUH-1528D30/2021|EPI_ISL_1806353|2021-04-21<br>hCoV-19/England/ALDP-152BE2B/2021|EPI_ISL_1806594|2021-04-21<br>hCoV-19/England/ALDP-152BE0D/2021|EPI_ISL_1806695|2021-04-21<br>hCoV-19/Scotland/MILK-152454B/2021|EPI_ISL_1806900|2021-04-18<br>hCoV-19/Scotland/MILK-152496D/2021|EPI_ISL_1806901|2021-04-20<br>hCoV-19/Scotland/MILK-15245B4/2021|EPI_ISL_1806902|2021-04-20<br>hCoV-19/Scotland/MILK-15243BA/2021|EPI_ISL_1806903|2021-04-18
**B.1.617.3**	hCoV-19/India/MH-NCCS-86945/2021|EPI_ISL_1415164|2021-02-13<br>hCoV-19/India/MH-NCCS-87388/2021|EPI_ISL_1415165|2021-02-16<br>hCoV-19/India/MH-NCCS-87005/2021|EPI_ISL_1415181|2021-02-14<br>hCoV-19/India/MH-NCCS-87272/2021|EPI_ISL_1415276|2021-02-16<br>hCoV-19/India/MH-NCCS-87400/2021|EPI_ISL_1415277|2021-02-16<br>hCoV-19/India/MH-NCCS-87017/2021|EPI_ISL_1415286|2021-02-14<br>hCoV-19/India/MH-NCCS-87447/2021|EPI_ISL_1415317|2021-02-16<br>hCoV-19/India/MH-NCCS-87273/2021|EPI_ISL_1415318|2021-02-16<br>hCoV-19/India/MH-NCCS-87387/2021|EPI_ISL_1415319|2021-02-16<br>hCoV-19/India/MH-NCCS-87434/2021|EPI_ISL_1415356|2021-02-16<br>hCoV-19/India/MH-NCCS-87412/2021|EPI_ISL_1415357|2021-02-16<br>hCoV-19/India/MH-NCCS-87026/2021|EPI_ISL_1415386|2021-02-14<br>hCoV-19/England/CAMC-143E11E/2021|EPI_ISL_1454202|2021-03-22<br>hCoV-19/India/ILSGS00782/2021|EPI_ISL_1663380|2021-03-02<br>hCoV-19/India/ILSGS00810/2021|EPI_ISL_1663409|2021-03-06<br>hCoV-19/India/ILSGS00820/2021|EPI_ISL_1663418|2021-03-08<br>hCoV-19/India/ILSGS00919/2021|EPI_ISL_1663495|2021-04-02<br>hCoV-19/India/ILSGS00975/2021|EPI_ISL_1663546|2021-03-13<br>hCoV-19/USA/CA-Stanford-15_S42/2021|EPI_ISL_1701680|2021-03-27<br>hCoV-19/India/MH-ICMR-NIV-INSACOG-GSEQ-193/2021|EPI_ISL_1704392|2021-02<br>hCoV-19/India/MH-ICMR-NIV-INSACOG-GSEQ-267/2021|EPI_ISL_1704407|2021-02-16<br>hCoV-19/India/MH-ICMR-NIV-INSACOG-GSEQ-467/2021|EPI_ISL_1704472|2021-03-02<br>hCoV-19/India/MH-ICMR-NIV-INSACOG-GSEQ-471/2021|EPI_ISL_1704474|2021-03-02<br>hCoV-19/India/MH-ICMR-NIV-INSACOG-GSEQ-733/2021|EPI_ISL_1704519|2021-03-03<br>hCoV-19/India/MH-ICMR-NIV-INSACOG-GSEQ-757/2021|EPI_ISL_1704526|2021-03-03<br>hCoV-19/India/MH-ICMR-NIV-INSACOG-GSEQ-764/2021|EPI_ISL_1704531|2021-03-15<br>hCoV-19/India/MH-ICMR-NIV-INSACOG-GSEQ-773/2021|EPI_ISL_1704535|2021-03-05<br>hCoV-19/India/MH-ICMR-NIV-INSACOG-GSEQ-1294/2021|EPI_ISL_1704623|2021-03-15<br>hCoV-19/England/ALDP-15192D9/2021|EPI_ISL_1759112|2021-04-17<br>hCoV-19/Russia/SPE-RII-32661S/2021|EPI_ISL_1797437|2021-04-21<br>hCoV-19/England/ALDP-153351C/2021|EPI_ISL_1830471|2021-04-23<br>hCoV-19/India/MH-NS1118/2021|EPI_ISL_1838050|2021-04-06<br>hCoV-19/India/MH-NS1119/2021|EPI_ISL_1838052|2021-04-06<br>hCoV-19/India/MH-NS1137/2021|EPI_ISL_1838097|2021-04-06<br>hCoV-19/India/MH-NS1152/2021|EPI_ISL_1838131|2021-03-15<br>hCoV-19/India/MH-NS1284/2021|EPI_ISL_1838345|2021-03-31<br>hCoV-19/India/AP-CCMB-BJ434/2021|EPI_ISL_1838377|2021-03-13<br>hCoV-19/India/AP-CCMB-BJ486/2021|EPI_ISL_1838380|2021-03-15<br>hCoV-19/India/AP-CCMB-BL326/2021|EPI_ISL_1838545|2021-03-26<br>hCoV-19/India/AP-CCMB-BL333/2021|EPI_ISL_1838552|2021-03-26

	hCoV-19/India/AP-CCMB-BN947/2021|EPI_ISL_1838655|2021-04-07<br>hCoV-19/India/TG-CCMB-BJ886/2021|EPI_ISL_1838772|2021-03-18<br>hCoV-19/India/MH-ICMR-NIV-INSAGOG-GSEQ-1377/2021|EPI_ISL_1928408|2021-03-06<br>hCoV-19/India/MH-ICMR-NIV-INSAGOG-GSEQ-1376/2021|EPI_ISL_1928411|2021-03-07<br>hCoV-19/India/MH-ICMR-NIV-INSAGOG-GSEQ-1246/2021|EPI_ISL_1928430|2021-03-14<br>hCoV-19/India/MH-ICMR-NIV-INSAGOG-GSEQ-1245/2021|EPI_ISL_1928432|2021-03-14<br>hCoV-19/India/MH-ICMR-NIV-INSAGOG-GSEQ-2167/2021|EPI_ISL_1928459|2021-03-23<br>hCoV-19/India/GJ-ICMR-NIV-INSACOG-GSEQ-2821/2021|EPI_ISL_1928510|2021-04-07<br>hCoV-19/India/MH-SEQ-221_S66_R1_001/2021|EPI_ISL_1939891|2021-03-22
**B.1.618**	hCoV-19/Turkey/HSGM-3219/2021|EPI_ISL_1117626|2021-01-28<br>hCoV-19/Singapore/275/2021|EPI_ISL_1312386|2021-03-17<br>hCoV-19/India/MH-1931500863416/2021|EPI_ISL_1419052|2021-02-19<br>hCoV-19/India/WB-1931300231694/2021|EPI_ISL_1419147|2021-02-17<br>hCoV-19/India/WB-1931200139612/2021|EPI_ISL_1419195|2021-02-04<br>hCoV-19/India/WB-1931300223894/2021|EPI_ISL_1419250|2021-02-02<br>hCoV-19/India/WB-1931300234499/2021|EPI_ISL_1419354|2021-02-22<br>hCoV-19/India/WB-1931300236872/2021|EPI_ISL_1419391|2020-10-26<br>hCoV-19/India/WB-1931300237620/2021|EPI_ISL_1419396|2020-10-27<br>hCoV-19/India/WB-1930300527247/2021|EPI_ISL_1419410|2021-02-22<br>hCoV-19/India/WB-1931300241740/2021|EPI_ISL_1419413|2021-03-07<br>hCoV-19/India/WB-1931300244017/2021|EPI_ISL_1419416|2021-03-12<br>hCoV-19/India/WB-1931500765852/2021|EPI_ISL_1419444|2021-01-21<br>hCoV-19/India/WB-1930400315726/2021|EPI_ISL_1419482|2021-01-28<br>hCoV-19/India/WB-1932000223806/2021|EPI_ISL_1419485|2021-02-16<br>hCoV-19/India/WB-1931500877129/2021|EPI_ISL_1419511|2021-02-23<br>hCoV-19/India/WB-1931500868167/2021|EPI_ISL_1419550|2021-02-20<br>hCoV-19/India/WB-1931500868122/2021|EPI_ISL_1419551|2021-02-20<br>hCoV-19/India/WB-1931500869429/2021|EPI_ISL_1419555|2021-02-20<br>hCoV-19/India/WB-1931500869442/2021|EPI_ISL_1419556|2021-02-20<br>hCoV-19/India/WB-1931500871419/2021|EPI_ISL_1419557|2021-02-21<br>hCoV-19/India/WB-1931500872957/2021|EPI_ISL_1419561|2021-02-22<br>hCoV-19/India/WB-1931500874921/2021|EPI_ISL_1419564|2021-02-22<br>hCoV-19/India/WB-1931500877555/2021|EPI_ISL_1419569|2021-02-23<br>hCoV-19/India/WB-1931500882376/2021|EPI_ISL_1419571|2021-02-23<br>hCoV-19/India/WB-1931500882322/2021|EPI_ISL_1419573|2021-02-24<br>hCoV-19/India/WB-1931500882398/2021|EPI_ISL_1419575|2021-02-24<br>hCoV-19/India/WB-1931500877383/2021|EPI_ISL_1419577|2021-02-24<br>hCoV-19/India/WB-1931500893199/2021|EPI_ISL_1419607|2021-02-27<br>hCoV-19/India/WB-1931500898749/2021|EPI_ISL_1419615|2021-03-01<br>hCoV-19/India/WB-1931500899362/2021|EPI_ISL_1419617|2021-03-01<br>hCoV-19/India/WB-1931500914459/2021|EPI_ISL_1419640|2021-03-05<br>hCoV-19/India/WB-1931500916033/2021|EPI_ISL_1419641|2021-03-05<br>hCoV-19/India/WB-1931500913500/2021|EPI_ISL_1419647|2021-03-04<br>hCoV-19/India/WB-1930300536764/2021|EPI_ISL_1419688|2021-02-26<br>hCoV-19/India/WB-1930300449497/2021|EPI_ISL_1419695|2021-01-14<br>hCoV-19/India/WB-1930300451753/2021|EPI_ISL_1419696|2021-01-15<br>hCoV-19/India/WB-1931500802025/2021|EPI_ISL_1419738|2021-02-01<br>hCoV-19/India/WB-1930300524189/2021|EPI_ISL_1419769|2021-02-20<br>hCoV-19/India/WB-194234878/2021|EPI_ISL_1419775|2021-02-13<br>hCoV-19/India/WB-1970400161836/2021|EPI_ISL_1419791|2021-02-20<br>hCoV-19/India/WB-1930600197769/2021|EPI_ISL_1419853|2021-02-17<br>hCoV-19/India/WB-1931800119269/2021|EPI_ISL_1419893|2021-03-02<br>hCoV-19/India/WB-1931800119273/2021|EPI_ISL_1419894|2021-03-02<br>hCoV-19/India/WB-1931500920028/2021|EPI_ISL_1419921|2021-03-06

	hCoV-19/Canada/ON-PHL-21-08963/2021|EPI_ISL_1559143|2021-03<br>hCoV-19/India/WB-1930300561111/2021|EPI_ISL_1589787|2021-03-09<br>hCoV-19/India/WB-1931300248145/2021|EPI_ISL_1589795|2021-03-20<br>hCoV-19/India/WB-1931500902089/2021|EPI_ISL_1589796|2021-03-02<br>hCoV-19/India/WB-1931300246768/2021|EPI_ISL_1589797|2021-03-17<br>hCoV-19/India/WB-1930400358832/2021|EPI_ISL_1589798|2021-02-21<br>hCoV-19/India/WB-1930400370165/2021|EPI_ISL_1589799|2021-02-24<br>hCoV-19/India/WB-1931500994932/2021|EPI_ISL_1589800|2021-03-25<br>hCoV-19/India/WB-1930300547970/2021|EPI_ISL_1589801|2021-03-03<br>hCoV-19/India/WB-1930300547898/2021|EPI_ISL_1589802|2021-03-03<br>hCoV-19/India/WB-1930300561592/2021|EPI_ISL_1589803|2021-03-09<br>hCoV-19/India/WB-1930300565789/2021|EPI_ISL_1589804|2021-03-11<br>hCoV-19/India/WB-1930300557101/2021|EPI_ISL_1589805|2021-03-07<br>hCoV-19/India/WB-1930300558684/2021|EPI_ISL_1589806|2021-03-08<br>hCoV-19/India/WB-1930300585540/2021|EPI_ISL_1589807|2021-03-19<br>hCoV-19/India/WB-1931500985650/2021|EPI_ISL_1589808|2021-03-23<br>hCoV-19/India/WB-1931700212203/2021|EPI_ISL_1589810|2021-03-04<br>hCoV-19/India/WB-1931700215417/2021|EPI_ISL_1589811|2021-03-11<br>hCoV-19/India/WB-1930400386726/2021|EPI_ISL_1589812|2021-03-09<br>hCoV-19/India/WB-1930400392771/2021|EPI_ISL_1589813|2021-03-12<br>hCoV-19/India/WB-1930400390662/2021|EPI_ISL_1589814|2021-03-11<br>hCoV-19/India/WB-1930400402838/2021|EPI_ISL_1589815|2021-03-19<br>hCoV-19/India/WB-1931500908129/2021|EPI_ISL_1589816|2021-03-03<br>hCoV-19/Germany/un-RKI-I-077252/2021|EPI_ISL_1642908|2021-04-07<br>hCoV-19/India/CT-ILSGS00421/2021|EPI_ISL_1662359|2021-02-09<br>hCoV-19/India/CT-ILSGS00422/2021|EPI_ISL_1662360|2021-02-09
